# Ruptured Caesarean Scar Ectopic Pregnancy: A Rare Case of Obstetric Hemorrhage

**DOI:** 10.7759/cureus.59422

**Published:** 2024-04-30

**Authors:** Gunjan Gunjan

**Affiliations:** 1 Obstetrics and Gynaecology, Patna Medical College, Patna, IND

**Keywords:** laparotomy, caesarean scar ectopic pregnancy, caesarean delivery, ectopic pregnancy, rupture scar ectopic

## Abstract

Caesarean scar ectopic pregnancy is the rarest form of ectopic pregnancy. Nowadays, with the rise in caesarean deliveries, along with better awareness and improvement in ultrasound diagnosis, there is an increase in the number and detection of caesarean scar ectopic pregnancy. A 28-year-old female patient with one previous caesarean delivery and a spontaneous abortion at three months visited the obstetrics emergency department due to three months of amenorrhea, abdominal pain, and vaginal bleeding on and off for two days. The patient was noticed to have severe anemia. After stabilizing the patient with blood transfusion, a laparotomy was performed with the presentation of hemoperitoneum and caesarean scar rupture. Fetus and soft vascular mass seen protruding from the previous scar were extracted. The caesarean scar site was repaired in layers.

## Introduction

Caesarean scar pregnancy is a rare form of ectopic pregnancy, occurring in one in 2000 pregnancies [1]. The rise of caesarean deliveries has increased the incidence of caesarean scar ectopic pregnancy [2]. The risk of caesarean scar ectopic pregnancy does not increase with the number of caesarean deliveries [3]. Disruption of the endometrium and myometrium after caesarean delivery causes improper implantations at the fibrous scar tissue and myometrium [4]. Untreated caesarean scar ectopic pregnancy may lead to uterine rupture with severe maternal hemorrhage and death [5]. Caesarean scar ectopic pregnancies mostly present in the first trimester. They are frequently misdiagnosed as normal intrauterine pregnancy, cervical pregnancy, and incomplete abortion. Here, we discussed a case of ruptured caesarean scar ectopic pregnancy. 

## Case presentation

A 28-year-old female patient with one previous caesarean delivery three years back and a history of spontaneous abortion at three months, not followed by dilatation and curettage, visited the obstetrics emergency department with a history of three months of amenorrhea with dull aching pain in the abdomen for one day which was aggravated by postural changes. She also complained of vaginal bleeding on and off for which she took treatment from a primary health center, but as her condition deteriorated, she was referred for the treatment of threatened abortion and severe anemia. The patient had a history of three months of amenorrhea and had taken antenatal checkups at the primary health center. There was no history of abortifacient intake or dilatation and curettage. Her earlier blood reports were normal, her previous scan showed a single, alive, intrauterine fetus of 11 weeks period of gestation. On examination, the patient was severely pale, ill-looking, and anxious. Her pulse was 130 bpm and her blood pressure was 90/60 mmHg. The patient's hemoglobin was 3.4 gm/dl. On abdominal examination, the abdomen was distended with the presence of guarding and rigidity. On paracentesis, blood was present. On bimanual examination, the cervical os was closed, and the uterus was mobile and anteverted with a size of 8-10 weeks. There was fornixes fullness on both sides, and cervical motion tenderness was present. Due to the suspicion of ectopic pregnancy or uterine rupture, the patient was immediately taken for laparotomy together with resuscitation and blood transfusion. After opening her abdomen, one liter of blood and clots were sucked out.

**Figure 1:**
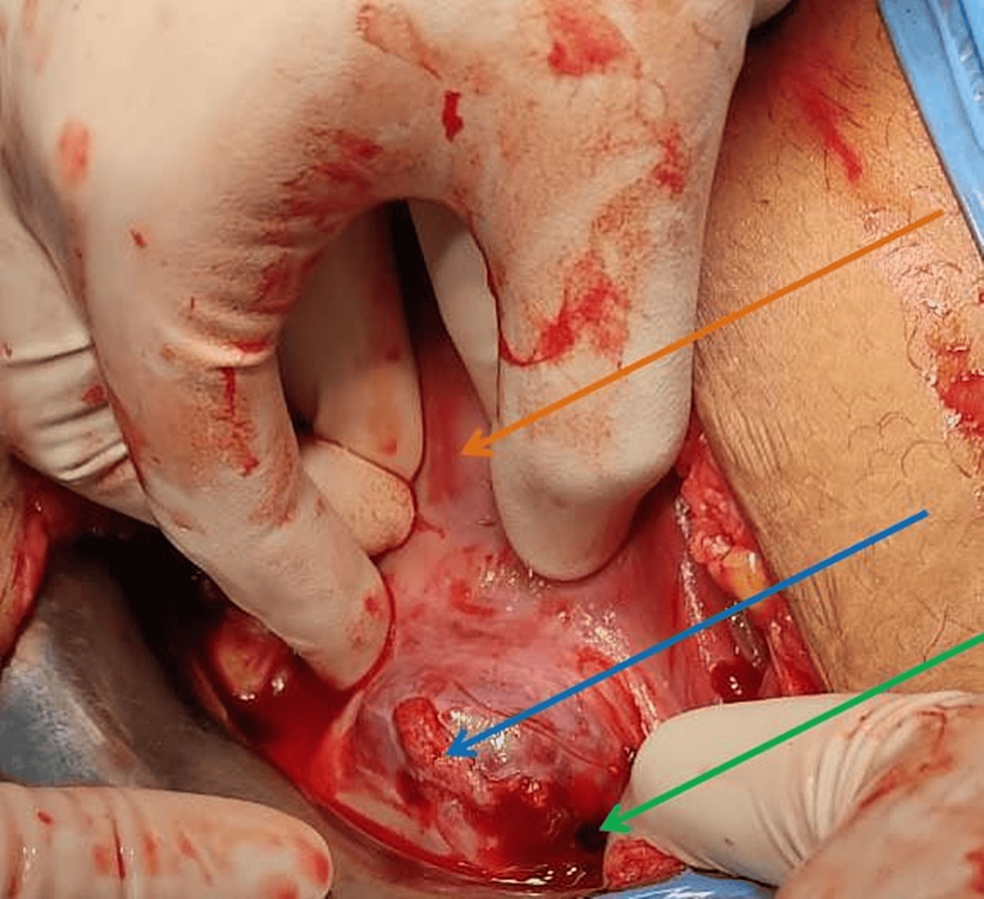
Caesarean scar ectopic pregnancy The orange arrow shows the empty uterine cavity, the blue arrow shows the scar ectopic pregnancy with a bulge at the lower uterine segment, and the green arrow shows the ruptured scar site

**Figure 2:**
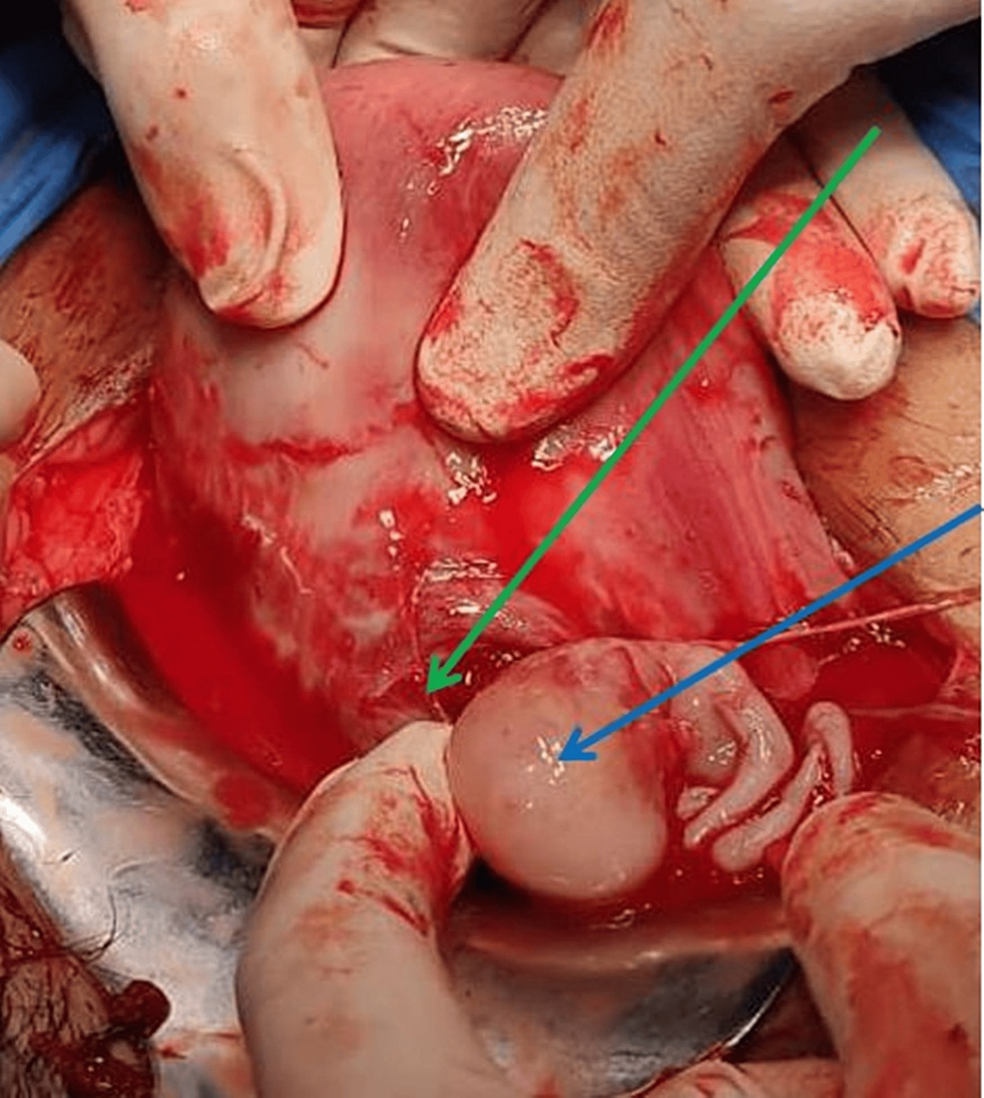
Ruptured scar ectopic pregnancy The green arrow shows the ruptured scar site, and the blue arrow shows the fetus being extracted from the scar site

**Figure 3:**
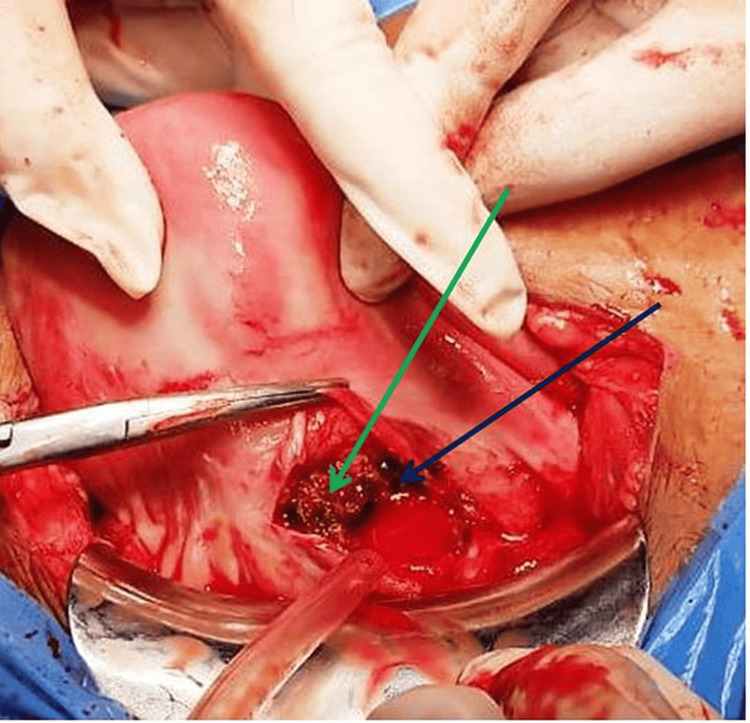
Ruptured scar ectopic with placenta The green arrow shows the placenta adherent to the previous scar site, and the blue arrow shows the ruptured caesarean scar

**Figure 4:**
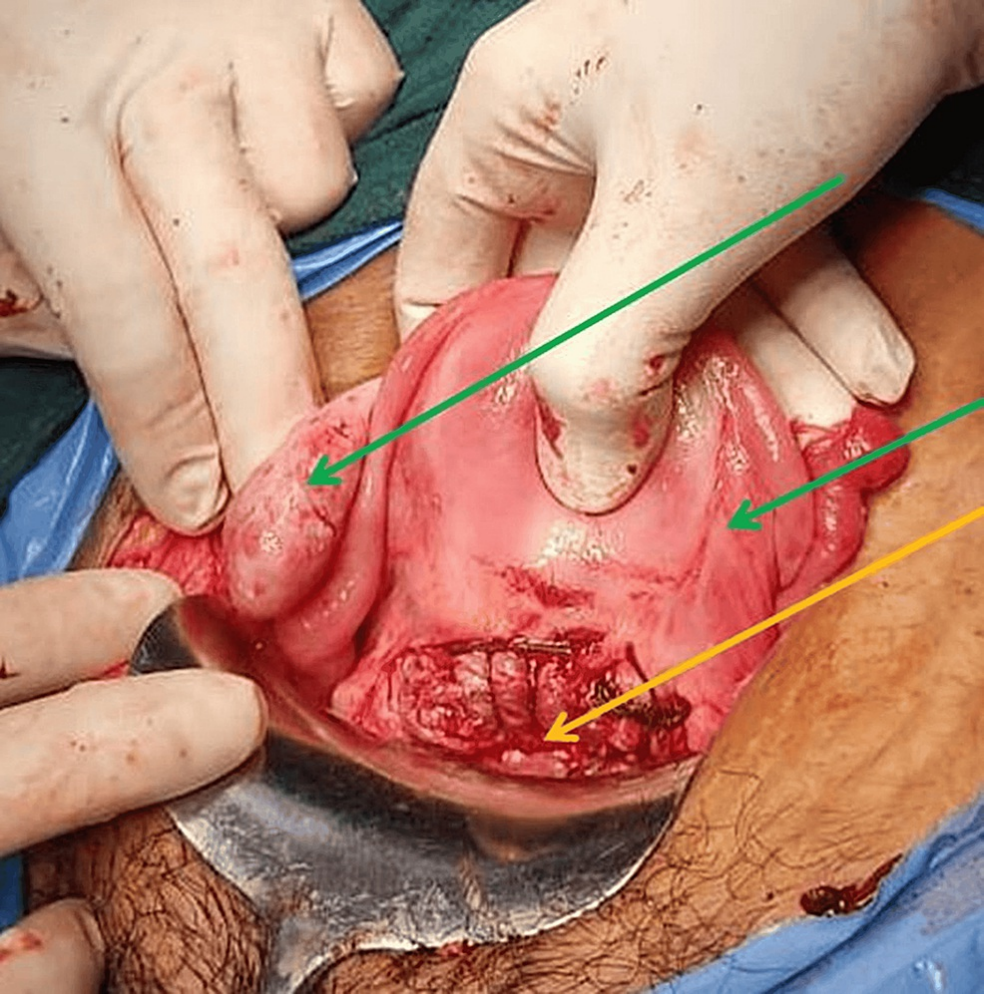
Uterus with the repaired scar of caesarean scar ectopic pregnancy The red arrow shows the repaired scar of ruptured caesarean scar ectopic pregnancy, and the green arrow shows both sides of tubes and ovaries

There was ballooning of the lower anterior scar as shown in Figure 1. There was a rupture of the caesarean scar on the left side of the uterus. The uterine cavity and the endocervical canal were also empty. The rupture site was extended, and a fetus of approximately 10-12-week gestational age was extracted from the caesarean scar site as shown in Figure 2. The placenta was adhered to the scar and removed in bits as shown in Figure 3. The previous scar site was resected and closed in layers after securing hemostasis as indicated in Figure 4. The patient was transfused four units of packed red blood cells and four units of fresh frozen plasma. Her postoperative period was uneventful. She was discharged in stable condition after stitch removal on her 10th postoperative day.

## Discussion

The presented case demonstrated a ruptured caesarean scar ectopic pregnancy which was managed by laparotomy with the scar repaired and the uterus preserved. A caesarean scar ectopic pregnancy is a long-term complication of caesarean section. Also, it is considered a rare ectopic pregnancy, and its incidence is increasing owing to the increasing rates of caesarean deliveries [6]. There are two types of caesarean scar ectopic pregnancy. In the first type, the gestational sac grows towards the cervico-isthmic space of the uterine cavity. Here, the pregnancy may continue till term but may lead to massive postpartum hemorrhage from the implantation site [7]. In the second variety, the gestational sac grows towards the serosal surface of the uterus. There is an increased risk of rupture in such cases during the first trimester.

In this case, the caesarean scar ectopic pregnancy presents with vaginal bleeding to uterine rupture and shock [8]. Any delay or misdiagnosis could lead to mismanagement and causes the rupture of caesarean scar ectopic pregnancy. In this case, delayed diagnosis led to the rupture of the scar, the patient landed in an emergency, and a life-saving laparotomy was done. In today's scenario, caesarean scar ectopic pregnancy can be managed in different ways. Early detection and diagnosis can be done by ultrasound. Treatment strategies include local and systemic injection of methotrexate, gestational sac aspiration, laparotomy, hysteroscopy, laparoscopy, and uterine artery embolization [3]. In our case, the patient was hemodynamically unstable due to the rupture of the scar so emergency laparotomy and excision of the ectopic pregnancy were done.

## Conclusions

There should always be a high degree of suspicion in treating patients with high-risk factors for scar ectopic pregnancy. Failure to diagnose can lead to serious complications. This case report study suggests exploring all possible sites of ectopic pregnancy during transvaginal scans, especially in high-risk cases. The radiologist should differentiate between spontaneous abortion and caesarean scar ectopic pregnancy. Caesarean scar ectopic pregnancy is a complicated condition with serious adverse results. Ultimately, the approach depends upon many factors like period of gestation, hemodynamic stability, hysteroscopy facilities, fertility plans, and feasible follow-ups.
